# The relationship between metacognitive beliefs and competitive anxiety among elite and amateur soccer players

**DOI:** 10.3389/fspor.2026.1880234

**Published:** 2026-07-22

**Authors:** Magnus Ueland Nygaard, Audun Havnen

**Affiliations:** 1 Norwegian Center for Sport and Mental Health, Oslo, Norway; 2 Department of Psychology, Norwegian University of Science and Technology, Trondheim, Norway; 3 Division of Psychiatry, St. Olav’s Hospital, Trondheim, Norway

**Keywords:** anxiety, competitive anxiety, metacognition, metacognitive beliefs, soccer (football)

## Abstract

**Background:**

Competitive anxiety is a commonly observed psychological phenomenon within the field of competitive sports such as soccer. Metacognitive beliefs are associated with anxiety in clinical and non-clinical populations, however, few studies have explored the relationship between metacognitive beliefs and competitive anxiety. In this study we aimed to explore the association between metacognitive beliefs and competitive anxiety among Norwegian soccer players. We also investigated differences in competitive anxiety between elite and amateur players, and between women and men.

**Methods:**

In a cross-sectional study, 150 Norwegian soccer players at elite and amateur level completed the Metacognitions Questionnaire 30 (MCQ-30), the Sport Anxiety Scale 2 (SAS-2), the Patient Health Questionnaire 9 (PHQ-9), and demographic variables. Independent *t*-tests were conducted to explore differences in competitive anxiety between elite and amateur and between female and male athletes. Hierarchical regression analyses were conducted to examine the relationship between metacognitive beliefs and total competitive anxiety scores, as well as the three SAS-2 subscales – somatic anxiety, worry and concentration disruption – while controlling for demographic variables and depressive symptoms.

**Results:**

Independent *t*-tests showed significantly higher competitive anxiety scores among amateur than elite players, and female players reported higher scores than male players. Regression analyses revealed that positive metacognitive beliefs were associated with somatic anxiety, while negative metacognitive beliefs and cognitive self-consciousness were associated with the worry subscale of SAS-2. Metacognitive beliefs were not related to the SAS-2 total score and concentration disruption.

**Conclusion:**

The results indicate a higher level of competitive anxiety among female and amateur players, and that metacognitive beliefs are relevant to somatic anxiety and worry among soccer players, irrespective of their competitive level. This suggests that interventions aimed at challenging metacognitive beliefs to reduce competitive anxiety should be explored in future studies. Theoretical and clinical implications of the current findings are discussed in light of the metacognitive model.

## Introduction

Competitive anxiety refers to an athlete's negative emotional reactions to stressful sport situations (1). Competitive anxiety has been conceptualized as a multidimensional construct that includes physiological components like somatic symptoms, and cognitive aspects like negative thoughts about performance (2). Cognitive anxiety has been further divided into the subcategories of worry and concentration disruption (3). The Sport Anxiety Scale 2 (SAS-2) (4) is one of the most frequently used measures of competitive anxiety. Studies show that competitive anxiety negatively affects performance. For example, anxiety is reported as the most important contributor to inadequate performance in soccer (5) and is associated with increased injury risk (6, 7). This highlights the need to identify factors that contribute to the development and maintenance of competitive anxiety.

Studies comparing competitive anxiety among elite athletes and recreational athletes have yielded mixed results. Some studies show equal levels of competitive anxiety across competitive levels (8), while other studies found higher competitive anxiety among less experienced athletes (9, 10). In addition, females and younger athletes tend to report higher competitive anxiety (11–13). The mixed results in previous studies may be due to differences in how experience levels have been operationalized, the measures used, and the types of sports studied. Age-related differences in competitive anxiety may reflect varying exposure to competitive stressors, where younger individuals are likely to have less experience in managing high-pressure situations. Some research suggests that elite athletes are more likely to perceive anxiety symptoms as beneficial for performance, indicating that they use more effective strategies for coping with pressure (1, 8). Further, amateur athletes with better coping strategies have been found to have an increased chance of progressing to higher levels (14).

The self-regulatory executive functioning (S-REF) model, often called the metacognitive model (15, 16) of psychological disorders, may offer a useful conceptual framework for understanding competitive anxiety. The model describes a cognitive attentional syndrome (CAS) characterized by maladaptive processing styles including worry and rumination, which maintain psychological symptoms like anxiety or depression. According to the model, the CAS is regulated by underlying metacognitive beliefs. These include positive beliefs about the perceived usefulness of worry (“Worrying helps me cope”), negative beliefs about the uncontrollability (“When I start worrying, I cannot stop”) or danger of worry (“Worrying will make me lose my mind”), and cognitive self-consciousness, which refers to monitoring one's thoughts and feelings (“I pay close attention to the way my mind works”) (16).

Studies systematically find that metacognitive beliefs are associated with symptoms of anxiety and depression (17), and dysfunctional metacognitions are identified across different mental disorders (18) as well as in the general population and student samples (19, 20). This highlights the transdiagnostic nature of the metacognitive model. Given the high comorbidity between anxiety and depression, and the evidence that metacognitive beliefs are strongly related to both types of symptoms, depression represents an important related construct to account for when examining the relationship between metacognitive beliefs and competitive anxiety.

The metacognitive model adds value beyond multidimensional theories of competitive anxiety, by shifting focus from the content of anxious thoughts to the processes that sustain them. Whereas traditional cognitive approaches typically target negative expectations about performance (e.g., “I might fail”) through cognitive restructuring, the metacognitive perspective conceptualizes such thoughts as part of the CAS, involving worry, threat-monitoring and maladaptive coping behaviors. Clinical practice therefore focuses on modifying underlying metacognitive beliefs about the usefulness or uncontrollability of these processes, rather than their specific content. This process-oriented focus offers an alternative pathway for reducing persistent anxiety by challenging the mechanisms that maintain it.

Because metacognitions may underlie multiple forms of psychological symptoms (18), exploring the association between metacognition and competitive anxiety within sports is particularly valuable. Despite the vast empirical literature on the metacognitive model, few studies have applied the model in sports contexts. A study including both team and individual athletes found that negative metacognitive beliefs were associated with higher cognitive and somatic anxiety (21). Research in triathletes similarly showed that negative beliefs about the uncontrollability and danger of worry and positive beliefs about worry were linked to increased worry before competition (22, 23), and that negative beliefs predicted somatic anxiety. Altogether, these results suggest that metacognitive beliefs may be important for the activation and maintenance of competitive anxiety in athletes. However, as the potential association between metacognitive beliefs and competitive anxiety in soccer players has received limited attention, more studies examining this topic are warranted.

In this study, we aimed to explore the association between metacognition and competitive anxiety in soccer players. The aims of the study were threefold: 1) compare the level of competitive anxiety measured with the SAS-2 across women and men, 2) examine whether the severity of competitive anxiety differs between athletes playing at elite or amateur level, and 3) explore the association between metacognitive beliefs and competitive anxiety, assessed through the SAS-2 global and subscale scores (somatic anxiety, worry, and concentration disruption), while controlling for relevant background variables (sex, age, competitive level, years of experience, health status, long-term illness and mental disorder) and depressive symptoms, which frequently co-occur with anxiety symptoms. We expected that women would report higher levels of competitive anxiety than men, and that athletes on elite level would show lower levels of competitive anxiety, compared to amateur athletes. Finally, we expected higher levels of dysfunctional metacognitive beliefs, especially positive and negative beliefs in accordance with previous research, to be associated with a higher degree of competitive anxiety.

## Methods

### Procedure

Participants were recruited by convenience by distributing information about the survey through social media and email lists within relevant soccer-based contexts, including the organization Norwegian Top Football and soccer podcasts. The survey was also distributed via the Norwegian Athlete's Association (NISO) to members of the soccer community. Information about the study was provided with a link to the survey, which was presented digitally through an electronic web-based survey portal. Participants were first introduced to the information sheet containing the purpose of the study, confidentiality, and rights as respondents. To proceed, participants first indicated informed consent by clicking a link presented below the information sheet, which forwarded them to the questionnaire. No compensation or payment was offered. Inclusion criteria were that participants had to be aged 16 years or older, be actively involved in organized soccer, and understand written Norwegian. No exclusion criteria were applied. According to the Norwegian Football Federation (NFF), the elite level consists of the two highest divisions among men and women and every division below these were categorized as amateur level.

The study was reported to the Regional Committees of Medical and Health Research Ethics (reference number 2024/737236). Participants remained anonymous as no personally identifiable information was gathered, but participants received the principal investigator's contact information in case they had any concerns about the survey.

### Participants

The sample consisted of 150 Norwegian soccer players between the age of 16–40 years (*M* = 24.62, *SD* = 5.74). Of these, 116 were male (77.3%) and 34 were female (22.7%). Across participants, 83 (55.3%) played at an elite level and 67 (44.7%) played at an amateur level. More than half of the participants (55.3%) had played on their respective level for more than five years, and most of the respondents considered their health as “good” (65.3%). Only a minority reported to have experienced a limiting longstanding illness (12.0%) or a history of mental disorder (8.7%). Table 1 shows background information of the sample.

**Table 1:** Descriptive statistics for demographic variables (*N* = 150).

Variable	*n*	*%*
Sex		
Female	34	22.7
Male	116	77.3
Level		
Elite	83	55.3
Amateur	67	44.7
Experience		
Up to 1 year	16	10.7
1–3 years	27	18.0
3–5 years	24	16.0
5 years or more	83	55.3
Health		
Very good	98	65.3
Good	51	34.0
Fair	1	0.7
Poor	0	0
Long-term illness		
Yes	18	12.0
No	132	88.0
Mental disorder		
Yes	13	8.7
No	137	91.3
	*M*	*SD*
Age	24.62	5.74
SAS-2 total score	26.69	7.98
SAS-2 somatic anxiety	8.24	2.92
SAS-2 worry	11.46	4.21
SAS-2 concentration disruption	6.99	2.42
PHQ-9	5.21	4.66
MCQ-30 POS	8.95	2.75
MCQ-30 NEG	10.06	3.56
MCQ-30 SCS	12.73	3.53
MCQ-30 NCT	10.30	2.92
MCQ-30 CC	8.49	3.28

PHQ-9, Patient Health Questionnaire-9; POS, positive beliefs about worry; NEG, negative beliefs about worry; CSC, cognitive self-consciousness; NCT, need to control thoughts; CC, cognitive confidence.

### Demographic variables

The respondents reported the following demographic variables: Sex (‘male’ or ‘female’), age, competitive level (‘elite’ or ‘amateur’), years of experience at one’s respective competitive level, health (‘very good’ ‘good’, ‘fair’, ‘poor’), limiting long-term illness (‘yes’ or ‘no’), and history of being diagnosed with a mental disorder (‘yes’ or ‘no’).

### Instruments

Metacognitions 30 (MCQ-30) (24) was used to measure metacognitive beliefs. The questionnaire consists of 30 items that are rated on a four-point Likert-scale from 1 (do not agree) to 4 (agree very much). Higher scores reflect stronger endorsement of metacognitive beliefs within the respective categories. The MCQ-30 measures five types of metacognitive beliefs: 1) positive beliefs about worry (“Worrying helps me avoid problems in the future”); 2) negative beliefs about worry (“My worrying thoughts persist, no matter how I try to stop them”); 3) cognitive self-consciousness (“I monitor my thoughts”); 4) need to control thoughts (“It is bad to think certain thoughts”); 5) cognitive confidence (“I have little confidence in my memory for places”). The MCQ-30 has good internal consistency and convergent validity, and good test-retest-reliability (24). Internal reliability in the current study was *α* = .87.

The Sport Anxiety Scale-2 (SAS-2) (4) was included to measure competitive anxiety. The SAS-2 has 15 items that are scored on a four-point Likert-scale from 1 (not at all) to 4 (very much). The instrument has three subscales: 1) somatic anxiety (“My body feels tense”), 2) worry (“I worry that I won't play well”) and 3) concentration disruption (“I lose focus on the game”). A global score, as well as total scores for each subscale, may be calculated, where higher scores indicate a more severe level of competitive anxiety. The scale is reliable, valid, and sensitive to change (3, 4). For this study, the SAS-2 was translated into Norwegian using a translation and back-translation approach. Cronbach's alpha in the current study was *α* = .92.

The Patient Health Questionnaire 9 items (PHQ-9) (25) was used to measure depressive symptoms. PHQ-9 assesses symptom severity by asking the respondents how often they have experienced a list of symptoms in the last two weeks from “not at all” (0) to “almost every day” (3). Total score range is 0-27, where higher scores indicate more severe symptoms. Based on total score, the symptom severity may be categorized as mild (5−9), moderate (10−14), moderately severe (15−19) and severe (20−27) (25). The internal reliability in this study was *α* = .88.

### Data analysis

An approximately normal distribution of scores was indicated through visual inspection of QQ-plots, PP-plots and histograms. The Variance Inflation Factor (VIF) indicated that multicollinearity was not a problem. To explore differences on a group level, independent *t*-tests were conducted to compare scores on SAS-2 between elite and amateur athletes, and between female and male athletes. Independent *t*-tests were conducted using two-tailed tests, as this represents a more conservative approach, despite directional hypotheses. Correlations between categorical variables were assessed with Spearman's *rho*, as it provides a rank-based, non-parametric estimate and allows for a consistent approach across variables, and continuous variables with approximately normal distributions were assessed using Pearson's *r*. Correlation coefficients below .40 were interpreted as weak, between .40-.69 as moderate, and .70 and above as strong (26).

To explore the relationship between metacognitive beliefs, demographic variables, depressive symptoms, and competitive anxiety, four stepwise hierarchical regression analyses were conducted. In these analyses, the total SAS-2 global score, and the subscales *somatic anxiety*, *worry* and *concentration disruption* were used as dependent variables, respectively. The four regression models contained identical steps. In step 1, the demographic variables, sex, age, competitive level, years of experience, health, long-term illness and history of mental disorder were entered. In step 2, depressive symptoms were included, and in step 3, the five metacognitive domains were entered. We regarded *p*-values below 0.05 to indicate statistical significance. The survey platform did not allow missing responses.

Participants were recruited using a convenience sampling approach. The sample size was considered sufficient for conducting the planned regression analyses, based on general recommendations regarding the number of observations per predictor variable (27).

## Results

### Group differences

Results of independent t-tests revealed significantly higher SAS-2 scores among amateur athletes (*M* = 28.69, *SD* = 9.00) than elite athletes (*M* = 25.07, *SD* = 6.68), *t*(118.87) =  −2.73, *p* < .01. Female athletes reported higher scores of SAS-2 (*M* = 33.53, *SD* = 7.42) than male athletes (*M* = 24.68, *SD* = 6.99), *t*(148) = 6.40, *p* < .001.

### Relationships between metacognitive beliefs and competitive anxiety

SAS-2 total score was moderately correlated with negative beliefs about worry and female sex and displayed a strong correlation with depressive symptoms. Negative metacognitive beliefs correlated most strongly with depressive symptoms. Correlation analyses are shown in Table 2.

**Table 2:** Correlations coefficients among study variables (*N* = 150).

Variables	2	3	4	5	6	7	8	9	10		11	12	13	14	15	16	17
1. SAS-2	.78^b^	.90^b^	.79^b^	.67^b^	.36^b^	.53^b^	.31^b^	.31^b^	.31^b^		−.50^b^	−.30^b^	.23^b^	−.12	.30^b^	−.14	−.24^b^
2. SAS-2-S		.53^b^	.50^b^	.59^b^	.32^b^	.43^b^	.21^a^	.24^b^	.30^b^		−.20^a^	−.13	.08	−.01	.23^b^	−.08	−.15
3. SAS-2-W			.60^b^	.52^b^	.31^b^	.48^b^	.32^b^	.26^b^	.23^b^		−.50^b^	−.30^b^	.27^b^	−.14	.27^b^	−.15	−.26^b^
4. SAS-2-C				.57^b^	.25^b^	.38^b^	.20^a^	.26^b^	.25^b^		−.43^b^	−.31^b^	.18^a^	−.14	.16^a^	−.11	−.23^b^
5. PHQ-9					.30^b^	.62^b^	.28^b^	.30^b^	.37^b^		−.34^b^	−.18^a^	.26^b^	−.06	.42^b^	−.26^b^	−.42^b^
6. POS						.31^b^	.35^b^	.38^b^	.15		−.20^a^	−.10	.14	−.15	.06	−.08	−.18^a^
7. NEG							.47^b^	.41^b^	.24^b^		−.26^b^	−.09	.16	−.03	.20^a^	−.17^a^	−.41^b^
8. CSC								.52^b^	.04		−.03	.09	.10	.02	.08	−.11	−.34^b^
9. NCT									.13		−.11	−.10	−.01	−.04	−.02	.07	−.09
10. CC											−.28^b^	−.11	−.01	.10	.17^a^	−.05	−.08
11. Female												.39^b^	−.25^b^	.13	−.10	−.00	−.12
12. Age													.10	.52^b^	−.07	−.07	.00
13. Elite														.12	.17^a^	−.04	−.25^b^
14. Experience															−.03	−.02	−.05
15. Health																−.15	−.16^a^
16. Illness																	.40^b^
17. Diagnosis																	

SAS-2, Sport Anxiety Scale-2 Total Score; SAS-2-S, Sport Anxiety Scale-2 – Somatic Anxiety; SAS-2-W, Sport Anxiety Scale-2 – Worry; SAS-2-C, Sport Anxiety Scale-2 – Concentration Disruption; PHQ-9, Patient Health Questionnaire-9; POS, positive beliefs about worry; NEG, negative beliefs about worry; CSC, cognitive self-consciousness; NCT, need to control thoughts; CC, cognitive confidence. Spearman's *rho* was used for correlations with ordinal or categorical variables, while Pearsońs *r* was used for continuous variables.

a *p* < .05.

b *p* < . 001.

For the regression analysis with SAS-2 total score as the dependent variable, the final model explained 57% of the variance. Male participants (*β* = −.19, *p* = .006) and older age (*β* = −.15, *p* = .048) were associated with lower levels of competitive anxiety. Inclusion of depressive symptoms significantly improved the model (*ΔR^2^* = .20, *p* < .001). Depressive symptoms were the strongest predictor (*β* = .44, *p* < .001) in the final model. The addition of metacognitive beliefs did not meaningfully improve the model, as reflected by the small increase in explained variance (*ΔR^2^* = .05).

For SAS-2 somatic anxiety, the final model explained 41% of the variance. Depressive symptoms (*β* = .55, *p* < .001) and positive metacognitive beliefs (*β* = .17, *p* = .030) were significant predictors in the final model.

For SAS-2 worry, the final model explained 49% of the variance. Male participants were associated with lower scores (*β* = −.29, *p* < .001). Negative metacognitive beliefs (*β* = .17, *p* = .048) and cognitive self-consciousness (*β* = .19, *p* = .021) were significantly associated with worry.

For SAS-2 concentration disruption, the final model explained 42% of the variance, with depressive symptoms as the strongest predictor (*β* = .47, *p* < .001). Male participants scored significantly lower than females (*β* = −.19, *p* *=* .019). Adding metacognitive beliefs did not improve the model (*ΔR^2^* = .01). A summary of the regression analyses is displayed in Table 3.

**Table 3:** Hierarchical regression analyses with the sport anxiety scale-2 as the dependent Variable (*N* = 150).

Step		Outcome																							
	Variable	SAS-2 total						SAS-2 somatic						SAS-2 worry						SAS-2 concentration disruption					
		*ΔF*	*R^2^*	*Adj.R^2^*	*ΔR^2^*	* β*	* t*	*ΔF*	*R^2^*	*Adj.R^2^*	*ΔR^2^*	* β*	* t*	*ΔF*	*R^2^*	*Adj.R^2^*	*ΔR^2^*	*β*	*t*	*ΔF*	*R^2^*	*Adj.R^2^*	*ΔR^2^*	* β*	* t*
1																									
	Sex					−.35	−4.35**					−.14	1.52					−.38	−4.94**					−.31	−3.69**
	Age					−.18	−2.00*					−.10	−.97					−.15	−1.73					−.20	−2.15*
	Level					.09	1.20					−.02	−.17					.14	1.88					.08	.96
	Exp					.02	.20					.07	.76					−.02	−.25					−.00	−.01
	Health					.19	2.60*					.20	2.37*					.18	2.50*					.07	.98
	Illness					−.08	−1.02					−.01	−.14					−.11	−1.41					−.06	−.73
	Diagnosis					−.11	−1.43					−.10	−1.10					.07	.84					−.14	−1.69
		9.61**	.32	.29	.32			2.22*	.10	.05	.10			11.14**	.36	.32	.36			7.01**	.26	.22	.26		
2																									
	PHQ−9					.57	7.69**					.67	7.82**					.33	4.03**					.51	6.19**
		59.19**	.52	.50	.20			61.08**	.37	.34	.27			16.24**	.42	.39	.07			38.27**	.42	.38	.16		
3	POS					.09	1.40					.17	2.19*					.05	.75					.01	.09
	NEG					.13	1.62					.12	1.28					.17	1.99*					−.02	−.18
	CSC					.12	1.66					.02	.21					.19	2.34*					.06	.67
	NC					−.00	−.06					−.05	−.55					−.01	−.09					.06	.66
	CC					.03	.47					.07	.90					.00	.03					.01	.20
		2.92*	.57	.53	.05			1.79	.41	.35	.04			3.85*	.49	.44	.07			.38	.42	.37	.01		

R^2^, total variance explained; Adj.R^2^, adjusted R^2^; *Δ*R^2^, increase in explained variance; Exp, years of experience; PHQ-9, Patient Health Questionnaire-9; POS, positive beliefs about worry; NEG, negative beliefs about worry; CSC, cognitive self-consciousness; NC, need to control thoughts; CC, cognitive confidence.

* *p* < .05.

** *p* < .001.

## Discussion

In this study we investigated differences in competitive anxiety between soccer players at elite and amateur levels and differences between women and men. We also explored the association between metacognitive beliefs and competitive anxiety. Amateur soccer players reported higher competitive anxiety than elite players, and women scored higher on competitive anxiety than men. Metacognitive beliefs were not associated with total competitive anxiety scores, while positive metacognitive beliefs were significantly associated with SAS-2 somatic anxiety*.* Further, negative metacognitive beliefs and cognitive self-consciousness were significantly associated with SAS-2 worry.

Consistent with expectations, amateur soccer players reported more competitive anxiety than elite players. However, it must be noted that part of the observed difference may reflect age effects rather than level alone, given the wide age range of the sample. Still, this finding is in line with previous studies that reported competitive anxiety to vary with age, with lower levels among more experienced athletes (9, 10). Several mechanisms may explain our findings. Elite athletes may be desensitized to competitive stress through repeated exposure to anxiety provoking situations. Athletes at higher levels may also receive more support that helps regulate anxiety. Amateur players may have less access to structured support than professionals and poorer strategies for coping effectively with heightened anxiety. Some studies find that elite athletes interpret anxiety as facilitative rather than harmful (8). Additionally, athletes with a high level of competitive anxiety may be less likely to reach elite levels due to impaired performances, although this should be investigated in prospective studies. However, it must be noted that previous studies have also yielded mixed results, where some studies found no differences in competitive anxiety between elite and non-elite athletes (8, 11).

We found that younger age was related to higher competitive anxiety. This corroborates previous studies that found younger athletes to report higher levels of distress and anxiety (11, 12). Possible explanations may be that younger athletes have encountered fewer competitions, and that with age and recurrent experience with competitive and anxiety provoking situations, athletes gradually become more skilled in applying appropriate strategies for regulating and tolerating sports-related stressors. Women reported higher levels of competitive anxiety. This corroborates previous studies showing higher anxiety symptoms in women, and further, that women often report anxiety to impact performance more negatively in both individual and team sports (12, 28, 29).

Contrary to expectations, there was no association between metacognitive beliefs and the SAS-2 total score. The reason for this may be that the global score reflects the three symptom domains worry, somatic anxiety and concentration disruption, which are distinct factors that differ in their potential relation to processes contained in the S-REF model. When these theoretically distinct domains are combined into the SAS-2 global score, associations with specific metacognitive processes may be reduced, which may explain why metacognitive beliefs were not significantly related to the total score. Negative metacognitive beliefs were significantly associated with SAS-2 worry. This finding is consistent with the metacognitive model, which emphasizes beliefs about the uncontrollability and danger of worry as central to maintaining worry processes (20, 30). The findings are partly in line with a study of triathletes (23) which found that negative beliefs, as well as positive beliefs, predicted cognitive anxiety.

Moreover, cognitive self-consciousness, which reflects a tendency to be preoccupied with internal thought processes, was associated with increased worry. It is possible that athletes who endorse this belief may become too focused on their internal dialogue during competition, which may in turn impair performance. According to the S-REF model, self-focused attention maintains the CAS and sustains worry, which may lead to increased insecurity during competition.

The results showed that positive metacognitive beliefs were associated with somatic anxiety. Athletes who believe that worrying is helpful (e.g., “worrying helps me cope”) may respond to physiological cues by worrying more, thereby increasing attentional focus on bodily sensations. Somatic anxiety symptoms may be amplified in turn. Increased internal focus has been shown to amplify the perception of bodily symptoms, a mechanism also observed in patients with health anxiety (31–33). Such attentional monitoring may intensify somatic anxiety during competition through worry and internal focus that backfire and increase bodily anxiety symptoms. Our findings differ from one previous study in which only negative beliefs predicted somatic anxiety (23), possibly due to methodological differences, such as the use of the CSAI-2 rather than the SAS-2 and the lack of adjustment for depressive symptoms.

Metacognitive beliefs were not related to the SAS-2 concentration disruption subscale. Within the S-REF framework, worry, rumination, maladaptive coping behaviors and threat monitoring are conceptualized as core components of the CAS. In contrast, the SAS-2 concentration disruption subscale reflects functional consequences of CAS processes during competition, such as disturbed attentional focus. Consequently, metacognitive beliefs may show stronger associations with SAS-2 subscales more closely related to CAS processes (i.e., worry and somatic anxiety) than with concentration disruption. However, it must also be noted that concentration disruption may be more strongly influenced by situational or performance-related influences, such as attentional demands during competition, which was not assessed in the present study.

Depression was the most consistent predictor in the regression analyses. Studies show a close relationship between metacognition, particularly negative metacognitive beliefs, and depression. Depression has also been associated with competitive anxiety among elite soccer players (34). When depressive symptoms were included in the regression model, the unique contribution of metacognitive beliefs to competitive anxiety was therefore reduced. This may indicate that depression accounts for variance that overlaps with metacognitive processes, which may mask associations between metacognitive beliefs and competitive anxiety. The strong association between depression and competitive anxiety should be considered both an important finding and a potential limitation of the study design. Still, metacognitive beliefs remained significantly associated with the SAS-2 subscales somatic anxiety and worry also when controlling for the effect of depression. Altogether, the different relationships between metacognitive beliefs and the SAS-2 subscales may indicate that metacognitive domains relate differently to specific components of competitive anxiety, which should be investigated further in future studies.

### Clinical implications

The relationship between metacognitive domains and the SAS-2 subscales worry and somatic anxiety suggests that principles proposed by the metacognitive model may be adapted to sports settings. The differences observed between amateur and elite players imply that interventions should be adapted to the athlete's level of experience. For coaches and practitioners, these findings suggest that attention should be directed towards challenging athletes’ beliefs about worry, or guiding athletes to disengage from bodily monitoring. Practical strategies may include brief psychoeducation about the limited usefulness of worry and encouraging task-focused attention during competition.

### Strengths and limitations

It is a strength that both amateur and elite soccer players were included, and that we used validated measures with sound psychometric properties. However, some limitations must be noted. The sample size was small and women were underrepresented, which limits gender comparisons. This also reduces the precision of estimates for women and increases the risk that results are influenced by outliers. Moreover, elite players were overrepresented in the sample, which could bias group comparisons. The wide age range in the sample (16−40 years) represents a limitation, as age and competitive experience are likely interrelated, and the observed differences between elite and amateur players may reflect differences in age rather than level of play. The cross-sectional design prevents any conclusion about cause and effect, and there is a risk of reverse causation, therefore, future studies should apply prospective designs. Given the well-known stigma of mental problems in sports settings, there is a risk of self-report bias and under-reporting symptoms. Lastly, all instruments were self-reported which represents a common methods bias.

## Conclusion

In line with the first hypothesis, we found that women reported higher levels of competitive anxiety than men. In support of the second hypothesis, we found that elite soccer players reported lower levels of competitive anxiety than amateur players. Hypothesis 3 was partially supported, as metacognitive beliefs were significantly associated with the SAS-2 subscales somatic anxiety and worry, but not with the SAS-2 total score and concentration disruption. Depressive symptoms were the most consistent predictor of competitive anxiety. The study provides support for the role of specific metacognitive beliefs in explaining competitive anxiety, specifically positive and negative metacognitive beliefs in addition to cognitive self-consciousness. Implications should be interpreted cautiously due to limitations of the current study, including the cross-sectional design, overrepresentation of male participants, uneven competitive level balance, and wide age range, which should be addressed in future research. The associations found in this study should be studied using prospective designs with more balanced samples including younger athletes and children, as early identification of maladaptive metacognitive beliefs may be important for preventing competitive anxiety.

## Data Availability

The raw data supporting the conclusions of this article will be made available by the authors, without undue reservation.
